# δ-Opioid Receptor Activation Modified MicroRNA Expression in the Rat Kidney under Prolonged Hypoxia

**DOI:** 10.1371/journal.pone.0061080

**Published:** 2013-04-15

**Authors:** Xiaozhou He, Yilin Yang, Feng Zhi, Meredith L. Moore, Xuezhi Kang, Dongman Chao, Rong Wang, Gianfranco Balboni, Severo Salvadori, Dong H. Kim, Ying Xia

**Affiliations:** 1 Research Institute of Modern Medicine, The Third Clinical College of Soochow University, Changzhou, Jiangsu, People’s Republic of China; 2 Department of Neurosurgery, The University of Texas Medical School at Houston, Houston, Texas, United States of America; 3 Laboratory of Molecular Neurology, Shanghai Research Center for Acupuncture and Meridians, Shanghai, People’s Republic of China; 4 Department of Pediatrics, Yale University School of Medicine, New Haven, Connecticut, United States of America; 5 Department of Life and Environment Sciences, University of Cagliari, Cagliari, Italy; 6 Department of Pharmaceutical Sciences, University of Ferrara, Ferrara, Italy; University of South Florida, United States of America

## Abstract

Hypoxic/ischemic injury to kidney is a frequently encountered clinical problem with limited therapeutic options. Since microRNAs are differentially involved in hypoxic/ischemic events and δ-opioid receptor (DOR) activation is known to protect against hypoxic/ischemic injury, we speculated on the involvement of DOR activation in altering the microRNA (miRNA) expression in kidney under hypoxic condition. We selected 31 miRNAs based on microarray data for quantitative PCR analysis. Among them, 14 miRNAs were significantly altered after prolonged hypoxia, DOR activation or a combination of both. We found that 1) DOR activation alters miRNA expression profiles in normoxic conditions; 2) hypoxia differentially alters miRNA expression depending on the duration of hypoxia; and 3) DOR activation can modify hypoxia-induced changes in miRNA expression. For example, 10-day hypoxia reduced the level of miR-212 by over 70%, while DOR activation could mimic such reduction even in normoxic kidney. In contrast, the same stress increased miR-29a by >100%, which was reversed following DOR activation. These first data suggest that hypoxia comprehensively modifies the miRNA profile within the kidney, which can be mimicked or modified by DOR activation. Ascertaining the targeted pathways that regulate the diverse cellular and molecular functions of miRNA may provide new insights into potential therapies for hypoxic/ischemic injury of the kidney.

## Introduction

Hypoxic/ischemic injury to kidney is a frequently encountered problem in vascular and urologic clinic given the sensitivity of kidney to changes in oxygen/blood delivery. Although blood flow to the kidney accounts for 20% of the cardiac output, the oxygen diffusion between arterial and venous vessels running parallel and in close proximity that keeps oxygen tension in the renal tissue comparatively low [1]. This high sensitivity to changes in oxygen tension makes the kidney prone to hypoxic/ischemic injury. Many clinical conditions, including chronic respiratory insufficiency, prolonged cardiovascular dysfunction, acute renal failure, chronic glomerular, tubulointerstitial or renovascular disease, diabetes, hypertension, aging, renal hypertrophy, anemia and obstructive uropathy, can reduce renal oxygenation and result in kidney dysfunction.

Hypoxia plays a significant role in the development of nephrotoxic acute kidney injury, radiocontrast nephropathy, and acute glomerulonephritis [1], [2]. In patients with chronic respiratory insufficiency, the arterial blood hypoxemia impairs the renal clearance of salt ions, urea and creatinine. The correlations between the renal clearances of these substances and the pO_2_ in arterial blood has a greater significance compared with the effects of pCO_2_ or [H^+^] levels, which in turn suggests the existence of a causal association between hypoxemia and renal dysfunction [3]. Severe energy depletion and subsequent activation of a number of critical alterations in metabolism and gene expression occur under hypoxic conditions. In fact, chronic hypoxia may be the final common pathway in end-stage kidney failure in kidney diseases. Hypoxic injury may have a pivotal role in both the development and progression of acute and chronic kidney diseases. Although hypoxic/ischemic insults are known to be associated with free radical induced injuries, the precise mechanisms underlying hypoxia-induced kidney injury remains poorly understood.

Recent research suggests that hypoxia, especially a prolonged stress, may influence the kidney through alterations in the RNAome. Over the past few decades, miRNAs have rapidly emerged as a new frontier in across different medical fields including kidney research [4], [5]. The kidney exhibits a unique miRNA expression profile [5]. Therefore, miRNA expression is likely important for renal function in normoxic condition and for the genesis of hypoxic pathology. Based on our previous studies [6]–[8], we hypothesized that hypoxia could influence the expression of miRNA in the kidneys. However, current literature on research studies involving this topic is scant.

There is no promising therapy available for the prevention and treatment of hypoxic kidney injury. We have recently demonstrated that the δ-opioid receptor (DOR) is protective against anoxic/ischemic injury in the brain and the underlying mechanisms involve the regulation of ionic homeostasis and antioxidative capacity [6], [7], [9]–[17]. In addition, DOR activation is also protective against hypoxic/ischemic injury in the heart [18]. In fact, studies show that DOR agonists can prolong survival of peripheral organs, such as lung, heart, liver, and kidney preserved en bloc or as a single preparation [19]. A growing body of evidence indicates that miRNAs play several regulatory roles in opioid pharmacology [20]–[22]. In view of this, we examined if DOR activation protects the kidney against hypoxic injury through a miRNA-mediated mechanism in the present study.

Our present study is a stepping stone in understanding hypoxic effect on the miRNAs in the kidney and its response to DOR activation in normoxia vs. hypoxia since there is no previously published data available on the subjects. Specifically, this work was conducted in adult rat kidney to determine: 1) if DOR activation alters miRNA expression profiles in the normoxic condition; 2) if short-term and prolonged hypoxia differentially alter miRNA expression; and 3) if DOR activation modifies hypoxia-induced changes in miRNA expression. The outcome of this study on renal miRNAs under hypoxic stress can result in discovery of new diagnostic biomarkers for kidney diseases. This in turn will provide novel insights into newer mechanisms of modulating the complex regulatory networks in the hypoxic kidney.

## Results

### Hypoxia-induced Changes in Body and Kidney Weights

**Figure 1:**
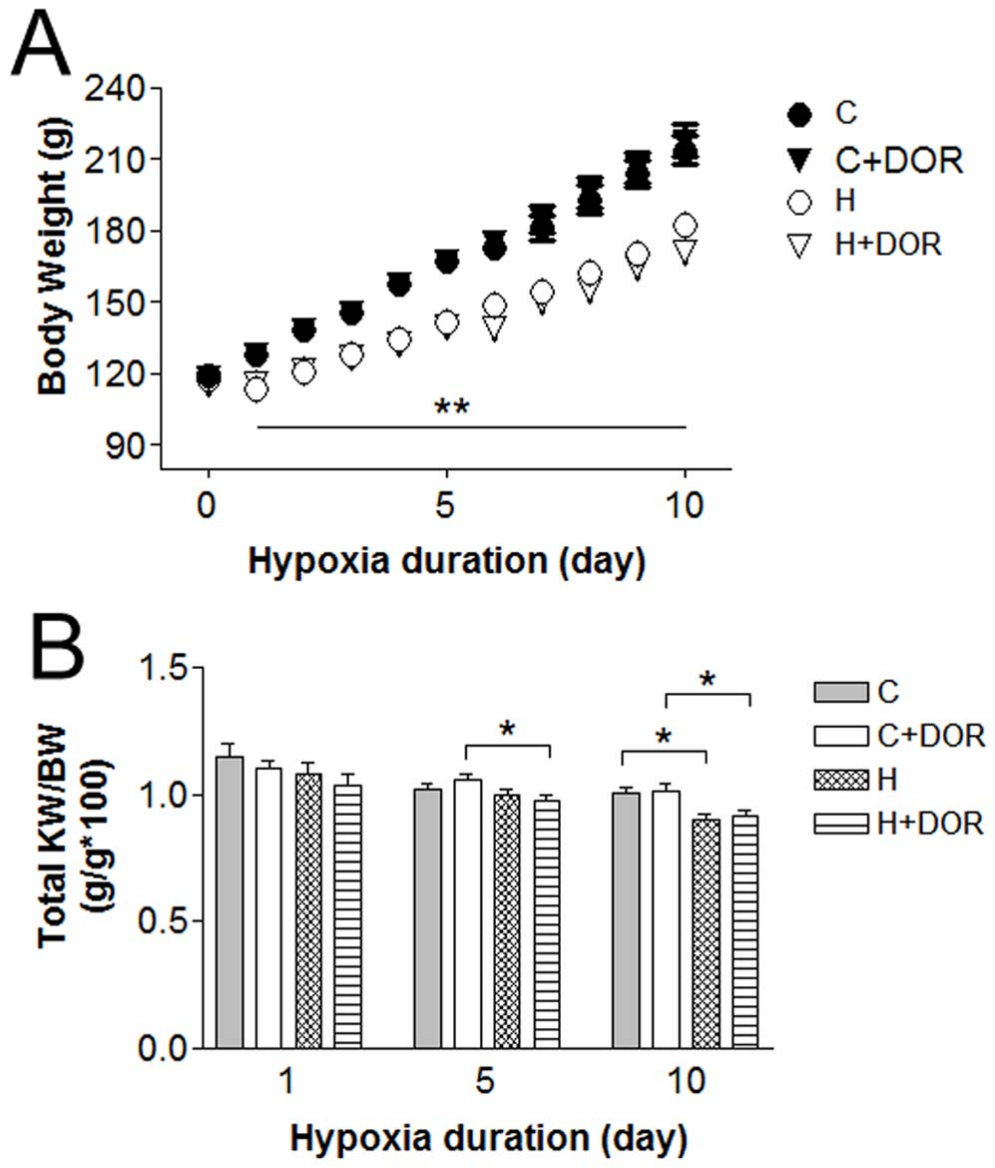
(A) Body weight measurement. Data points represent the mean ± SE with a minimum n = 9. C, control; C+DOR, control+UFP-512; H, hypoxia; H+DOR, hypoxia+UFP-512. **p<0.01 (C vs. H; C+DOR vs. H+DOR) for all time points encompassed by the bar. (**B**) Kidney weight/body weight ratios for each study group. *p<0.05, **p<0.01 with student’s t-test.

Changes in body and kidney weight were monitored in all animals. Hypoxic rats demonstrated a reduced ability to gain weight that was significant after 24 hours of hypoxic treatment and persisted for the remainder of the experiment (Figure 1a). Individual kidney weights were also significantly lower in the hypoxic animals (data not shown). The addition of UFP-512, a DOR agonist, did not affect the rate of increase in body weight neither in the control nor the hypoxic groups. The ratios of kidney/body weight were significantly lower following chronic hypoxia indicating that the kidneys exhibited a greater sensitivity to oxygen deprivation (Figure 1b). This reduction in kidney/body weight ratio was recorded earlier in the UFP-512 treated animals and was statistically significant as early as 24 hours after hypoxia (Figure 1b).

### MicroRNA Microarray Analysiss

**Figure 2:**
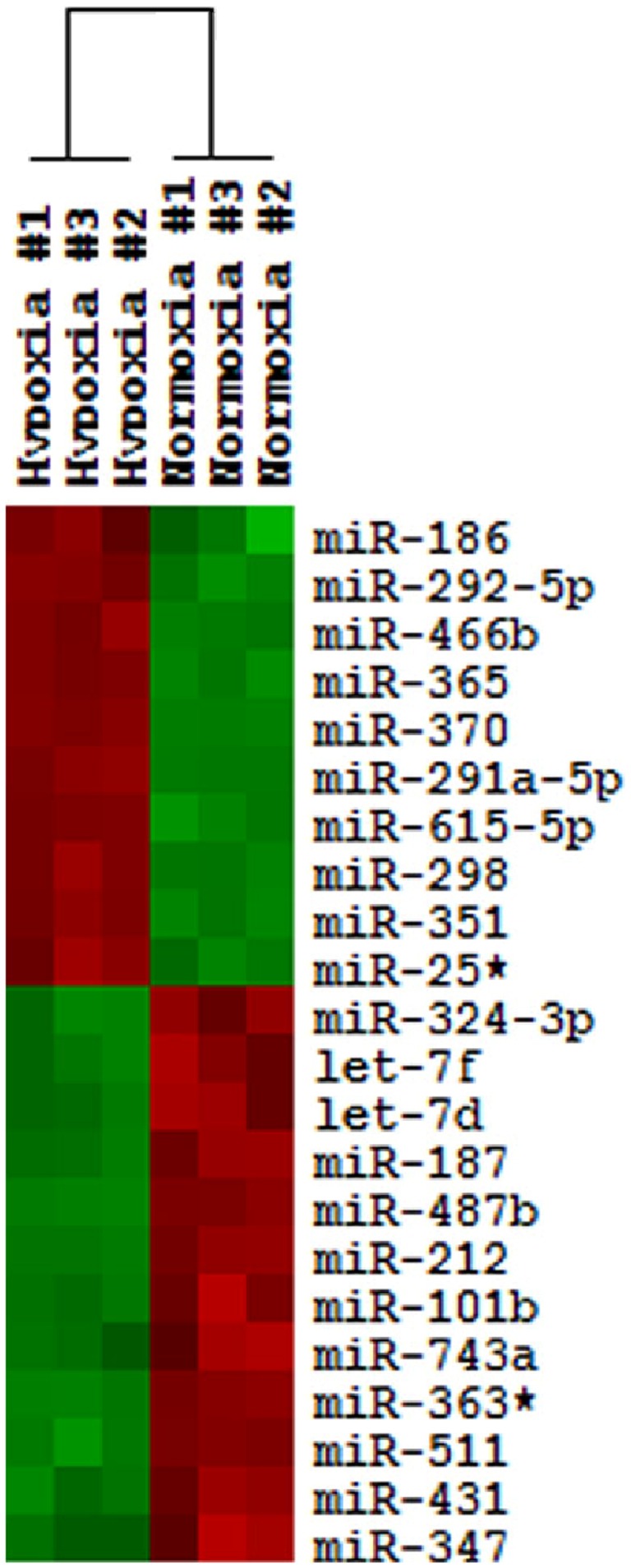
Dendrogram of aberrant miRNA expression in the kidney after hypoxia. Unsupervised hierarchical cluster analysis of 22 miRNAs differentially expressed in the kidney after hypoxia separates hypoxic kidney from control samples based on miRNA profiling. Upregulated miRNAs are labeled red and downregulated miRs are colored green.

To identify the molecular changes induced by hypoxia, comparative miRNA microarray analysis was performed on RNA isolated from the kidneys of rats exposed to 10-day hypoxia and normoxia. Analysis revealed that 22 miRNAs were significantly and differentially expressed between hypoxic and control samples (Figure 2). Of these 22 subtypes of miRNAs known to be expressed in the rat kidney, 12 were significantly downregulated, while 10 were significantly upregulated. All these changes in 22 miRNAs were verified by RT-PCR. In addition to the miRNAs selected following microarray analysis, we also investigated supplementary miRNAs which were proven to be correlated with hypoxia or ischemia-responsiveness in other organs. In total, 31 miRNAs were selected for further quantitative PCR analysis, which include let-7d, let-7f, miR-25*, miR-101b, miR-186, miR-187, miR-212, miR-291a-5p, miR-292-5p, miR-298, miR-324-3p, miR-347, miR-351, miR-363*, miR-365, miR-370, miR-431, miR-466b, miR-487b, miR-511, miR-615-5p, miR-743a, miR-20b-3p, miR-20b-5p, miR-21, miR-29a, miR-29b, miR-31, miR-135a, miR-199a-3p and miR-199a-5p [23]–[28].

### Hypoxia and DOR Activation Shifted miRNA Expression Profiles of Rat Kidney

**Figure 3:**
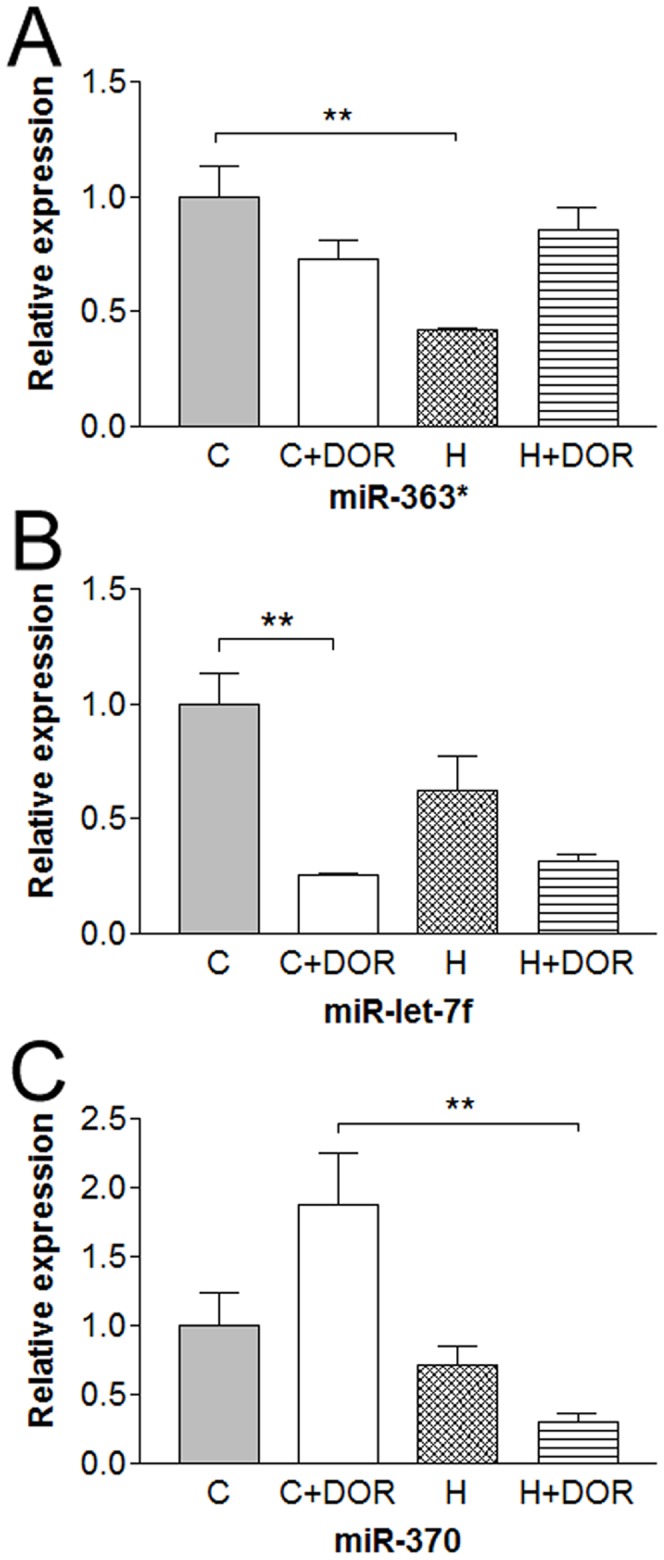
Relative miRNA expression levels of miR-363*, miR-let-7f, and miR-370 in the kidney following 10 days of hypoxia as determined by quantitative RT-PCR. C, control; C+DOR, control+UFP-512; H, hypoxia; H+DOR, hypoxia+UFP-512. **p<0.01.

Quantitative RT-PCR analysis confirmed the direction and amplitude of all miRNA changes with the exception of let-7d, miR-25*, miR-187, miR-291a-5p, miR-292-5p, miR-365, miR-431, miR-487b, miR-615-5p, miR-743a, miR-20b-3p, miR-199a-3p which remained unaltered or showed no statistical significance. A subset of miRNAs was influenced only by long-term exposure to hypoxia or DOR agonist. Chronic hypoxia for 10 days depressed miR-363* expression greater than 50%. DOR treatment alone did not significantly alter basal miR-363* levels in the normoxic kidney, but prevented hypoxia-induced miR-363* down-regulation (Figure 3a). In contrast, the same hypoxic exposure did not induce any significant reduction in let-7f despite the tendency to decrease let-7f under hypoxia (Figure 3b). However, reduced let-7f levels were noted in the kidneys following long-term DOR agonist exposure (Figure 3b). As with let-7f, miR-370 did not alter in response to hypoxia alone. Conversely, UFP-512 tended to increase miR-370 in normoxic condition but tended to decrease it under hypoxia (Figure 3c), suggesting a differential regulation of miR-370 in response to DOR signals in different environments.

**Figure 4:**
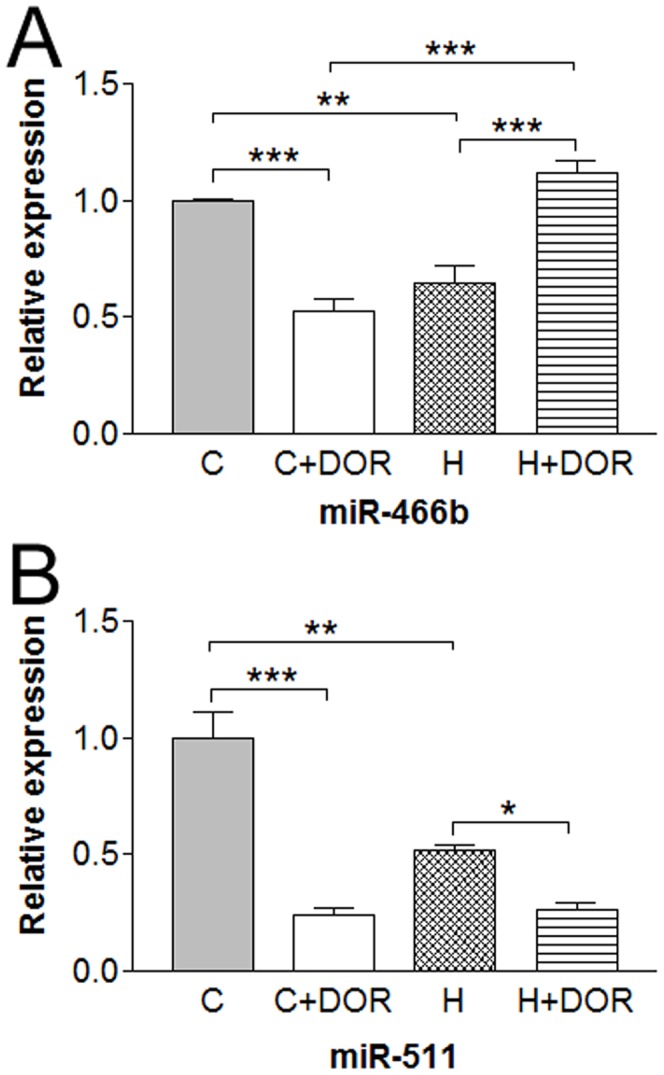
Relative miRNA expression levels of miR-466b and miR-511 in the kidney following 10 days of hypoxia as determined by quantitative RT-PCR. C, control; C+DOR, control+UFP-512; H, hypoxia; H+DOR, hypoxia+UFP-512. *p<0.05, **p<0.01, ***p<0.001.

The miRNA-466b levels were significantly repressed by either long-term hypoxia or UFP-512 treatment. Interestingly, the combination of long-term hypoxia and UFP-512 treatment returned miR-466b expression to control levels (Figure 4a). The miR-511 levels were also down-regulated by chronic hypoxia. The addition of UFP-512 further downregulated miR-511 levels in the hypoxic kidney when compared to DOR treatment with UFP-512 alone (Figure 4b).

### Longitudinal miRNA Changes in Response to Hypoxia

**Figure 5:**
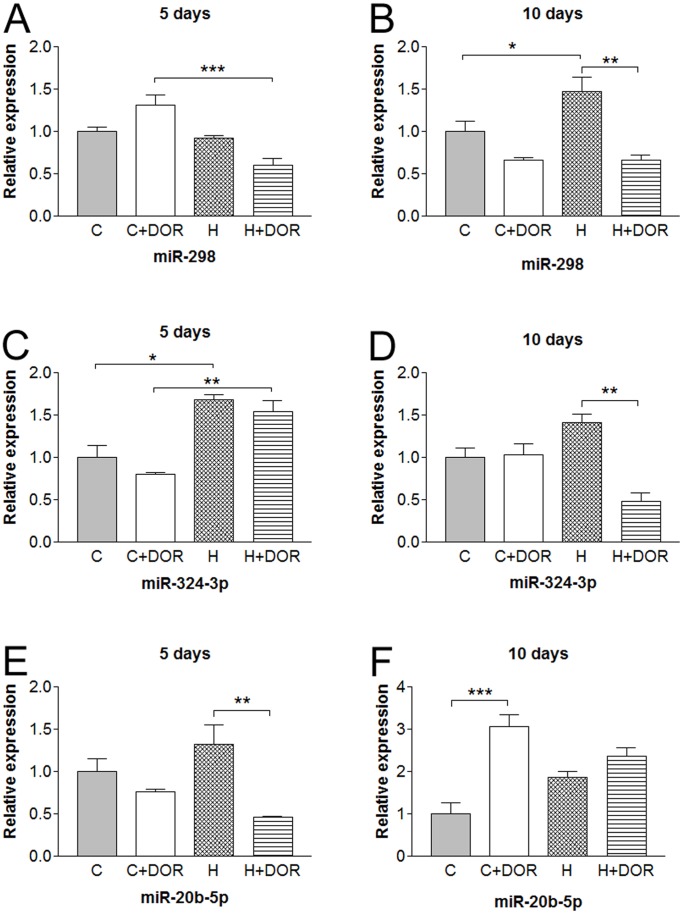
Relative miRNA expression levels of miR-298, miR-324-3p, and miR-20b-5p in the kidney following either 5 or 10 days of hypoxia as determined by quantitative RT-PCR. C, control; C+DOR, control+UFP-512; H, hypoxia; H+DOR, hypoxia+UFP-512. *p<0.05, **p<0.01, ***p<0.001.

Several miRNAs were altered at two given time points in the least. In the presence of UFP-512, miR-298 showed a hypoxia-induced downregulation that was lost after 10 days of hypoxia. Hypoxia alone did significantly upregulate miR-298 expression by 50% at 10 days while DOR treatment returned the levels to baseline. The miRNA 324-3p was consistently upregulated by hypoxia with or without DOR signaling following 5-day treatment. After 10 days of treatment, the upregulated miR324-3p expression seen in 5-day hypoxia disappeared and was further downregulated by DOR treatment in hypoxia. A similar DOR-induced downregulation in the presence of hypoxia was also found in miR-20b-5p at 5-day time point, which was disappeared at 10 days of hypoxia but replaced by a 3-fold stimulation of miR-20b-5p expression with UFP-512 treatment (Figure 5).

**Figure 6:**
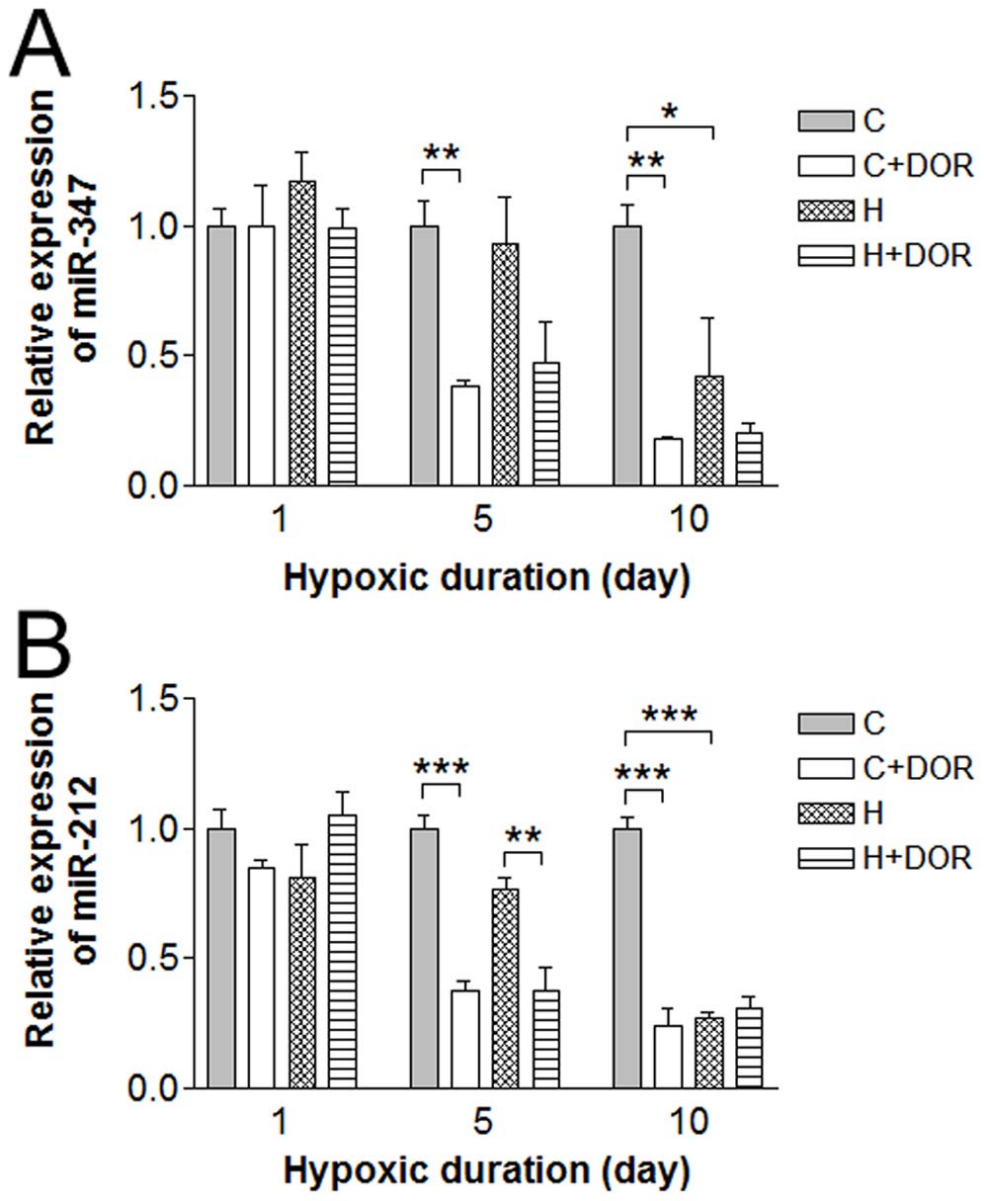
Relative miRNA expression levels of miR-347 and miR-212 in the kidney following either 1, 5, or 10 days of hypoxia as determined by quantitative RT-PCR. C, control; C+DOR, control+UFP-512; H, hypoxia; H+DOR, hypoxia+UFP-512. *p<0.05, **p<0.01, ***p<0.001.

The miR-347 and -212 expression showed no appreciable effect following exposure to hypoxia for 1-day and/or DOR activation, whereas a longer duration (5 or 10 days) of treatment demonstrated marked alterations in their levels of renal expression (Figure 6). While hypoxia alone had no effect on miR-347 expression after 5 days, UFP-512 treatment produced a 60% reduction in normoxic condition, and induced a similar change under hypoxia. The DOR-triggered depression was further increased to 25% of the control level after 10 days. DOR activation did not alter these hypoxic changes significantly (Figure 6a). In 5-day samples, miR-212 was largely reduced by UFP-512 in both the control and hypoxic conditions. Hypoxia alone did not significantly affect miR-212 expression until the 10-day time point. With this chronic hypoxia, miR-212 levels were repressed to less than 40% of the control in all combinations of hypoxia and UFP-512 treatment tested (Figure 6b).

**Figure 7:**
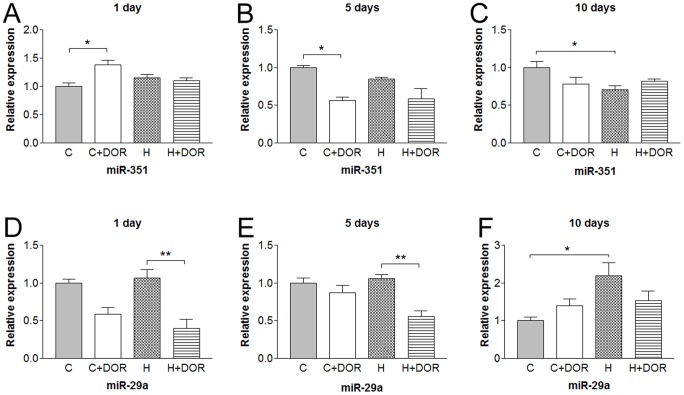
Relative miRNA expression levels of miR-351 and miR-29a in the kidney following either 1, 5, or 10 days of hypoxia as determined by quantitative RT-PCR. C, control; C+DOR, control+UFP-512; H, hypoxia; H+DOR, hypoxia+UFP-512. *p<0.05, **p<0.01.

Four miRNAs investigated were altered by hypoxia and/or DOR activation at all three time points. In the case of miR-351, 10-day hypoxia induced a 30% decrease though no significant reduction was seen after 1- or 5-day hypoxia. The miR-351 expression after UFP-512 treatment showed an initial slight yet significant upregulation following 1-day treatment, followed by a substantial downregulation by 45% after 5 days. Chronic UFP-512 treatment did not alter miR-351 levels under hypoxia. Hypoxia alone downregulated the miR-351 expression after 10 days of exposure (Figure 7). Although UFP-512 treatment alone did not alter miR-29a levels, the addition of UFP-512 to the hypoxic kidney significantly downregulated the miR-29a expression to 40–60% of the control levels after both 1 and 5 days of hypoxia. Following chronic exposure to hypoxia, miR-29a was upregulated by more than 100% as opposed to hypoxia alone. The addition of UFP-512 appeared to reduce the hypoxia-increased level of miR-29a (Figure 7).

**Figure 8:**
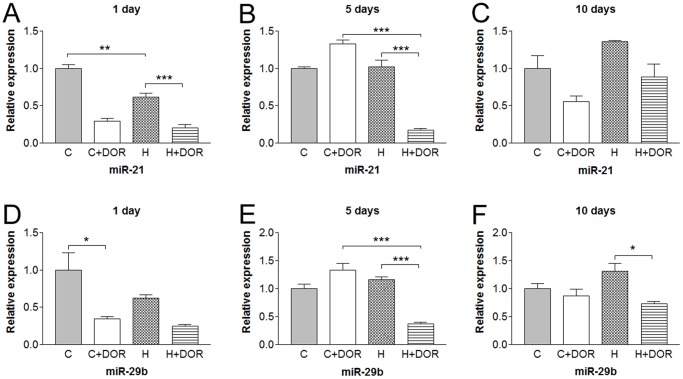
Relative miRNA expression levels of miR-21 and miR-29b in the kidney following either 1, 5, or 10 days of hypoxia as determined by quantitative RT-PCR. C, control; C+DOR, control+UFP-512; H, hypoxia; H+DOR, hypoxia+UFP-512. *p<0.05, **p<0.01, ***p<0.001.

Hypoxia for 1 day reduced miR-21 expression by 40% and the addition of UFP-512 further reduced its expression to 25% of the control levels (Figure 8). After 5 days the hypoxia-induced reduction disappeared, but the combination of hypoxia and UFP-512 produced a dramatic loss of miR-21. The presence of UFP-512 alone suppressed miR-29b levels following 1 day of hypoxia exposure (Figure 8). Either hypoxia or UFP-512 alone had no effect of miR-29b at 5 days, but the combination significantly stunted miRNA levels similar to miR-21. After 10 days, DOR stimulation significantly depressed miR-29b levels only in the presence of hypoxia.

## Discussion

Current research on describing the renal miRNA profiles focusses on neoplastic lesions or cultured fibroblasts. Here we present the first data that identify 14 miRNAs in the intact kidney altered in response to chronic hypoxia and DOR activation. Our comprehensive data demonstrate that 1) DOR activation shifts miRNA expression profiles in normoxic conditions; 2) hypoxia differentially alters the miRNA expression profiles depending hypoxic duration; and 3) DOR activation modifies hypoxia-induced changes in miRNA expression, suggesting significant hypoxia- and DOR-induced alterations in the miRNA profiles of the kidney.

There is accumulating evidence on the diagnostic and therapeutic potentials of a new class of small regulatory RNA molecules, termed microRNAs. These miRNAs are a class of endogenous non-coding RNAs comprised of 19–24 nucleotides that regulate the post-transcriptional silencing of protein-coding genes by base-pairing to complementary sites on their target mRNAs, thus suppressing protein synthesis, promoting protein degradation or translational blockade. Several miRNAs (e.g., miR-29) that showed a dynamic change with hypoxic durations in this work regulate the process of oxidative stress and inflammation and are involved in regulation of apoptotic and survival signal pathways,. The miR-29 family directly target at least 16 extracellular matrix genes and are relevant to renal and cardiovascular injury [29]–[31]. Recent research suggests that miR-29 inhibits cell proliferation and induces cell cycle arrest [32], modulates oxidative injury [33] and promote apoptosis through a mitochondrial pathway that involves Mcl-1 and Bcl-2 [28]. We observed that 1-day hypoxia down-regulated miR-29a expression, which is probably an adaptive compensation of the kidney for overcoming the hypoxic stress. However, the kidney loses this ability to compensate after a prolonged period (5 days) of hypoxia. Furthermore, hypoxic exposure for an even longer duration, i.e., 10 days, significantly increased both miR-29a and miR-29b levels that can explain induction of renal injury under prolonged hypoxia. Interestingly, DOR activation decreases the level of miR-29a in all these scenarios, suggesting a potential for therapeutic utilization of DOR activation in hypoxic/ischemic renal injury.

A few of the miRNAs showed changes only after 10 days of hypoxia. For example, chronic hypoxia largely reduced expression of miR-363*. DOR activation prevents the hypoxic reduction of miR-363*, suggesting that DOR signaling may regulate miR-363*, thus reducing hypoxic/ischemic injury. In contrast, DOR activation demonstrated an opposite effect on other miRNAs. For example, when hypoxia exposure decreased miRNA let-7f, DOR activation failed to increase the level of let-7f. Instead, it further potentiated the let-7f reduction under hypoxia, which suggests a differential regulation of various miRNAs through DOR activated pathways in the hypoxic kidney.

Our longitudinal study identified three DOR-sensitive miRNAs (miR-347, -212 and -351) that were downregulated after 5 days way ahead in time when the same change was induced by hypoxia after 10 days of exposure. These three miRNAs are predicted to target the scaffolding protein Bassoon, involved in vesicle clustering and regulate trans-Golgi trafficking in the hypoxic kidney. In a study on alcoholic liver disease in which alcohol-induced gut leakiness is a key factor, ethanol increased miR-212 expression, decreased the level of Zonula occludens 1 protein, disrupted tight junctions, and increased the permeability of monolayers of Caco-2 cells [34]. This study suggests an adverse effect of increased miR-212 on tissues in pathophysiological conditions. Therefore, the hypoxic reduction of miR-212 may be an adaptive strategy of the kidney in response to hypoxia. The notion that DOR action could mimic the hypoxic change in miR-212 provides additional evidence since DOR activation is protective against hypoxic injury [6]–[8], [16]. Also, these miRNAs are predicted to target the HIF-regulator MAF and prolong the expression of this hypoxia-specific factor. Accumulated HIF binds to the hypoxia-responsive element within the erythropoietin promoter and activates transcription of erythropoietin to increase erythropoietin production [1]. Erythropoietin has been proven as a hypoxia-responsive cytokine to provide protective effects in the damaged brain during hypoxic/ischemic events and neurodegenerative diseases [35], [36]. Therefore, the above-mentioned changes may potentially induce a protective effect on the hypoxic kidney.

The downregulation of miR-21 and miR-29b produced by either hypoxia or DOR activation in 1-day group appeared to be reversed by day 5 unless the two treatments (hypoxia plus DOR activation) were given simultaneously. Addition of UFP-512 suppresses these miRNAs and enhances the expression of a subset of mRNAs targeted by miR-21 and miR-29b. Expression of these genes is important for the structure of the basement membrane and kidney development, cell signaling as well as the matrix and junctional proteins matrilin and vinculin. Our data suggest that their expression could be modified during hypoxia with the addition of DOR agonists and thus affect the survival of the kidney in hypoxic environment. DOR is known to prevent hypoxic disruption of Na^+^ and K^+^ homeostasis in the brain through a PKC-dependent pathway [12]–[15], [17]. DOR activation may regulate ionic homeostasis partially via miR-21 since PKCε and several K^+^ channels are predicted targets of miR-21. Recent evidence also suggests the miR-21 inhibition of the PKC substrate MARCKS [37]. On the other hand, DOR activation to prolong miR-29b suppression could lengthen the expression of HIF3a as well as its transcriptional regulators MAFG and MAFB.

Proteinuria, resulting from a disrupted slit diaphragm, is an indication of kidney injury. Recently, the presynaptic adhesion molecule, neurexin-1, was reported to be present in the podocytes of the glomerular epithelial cells forming this capillary wall barrier in the kidney [38]. Models of kidney injury demonstrate a loss of neurexin and propose a role for the transmembrane protein in the formation and maintenance of the kidney slit diaphragm [39]. Neurexophilin, a ligand for neurexin, is one of the predicted targets of the hypoxia-upregulated miR-298. Prolonged hypoxia (10 days) significantly increased the level of miR-298, suggesting a downregulation of Neurexophilin. This alteration could potentially reduce the functionality of the neurexin signaling pathway, which may mimic the proteinuria/injury seen in other models of kidney disease and contribute, at least partially, to kidney damage in prolonged hypoxia. The application of DOR agonist attenuated these changes in miR-298 expression. In addition, the downregulation of miR-511 seen at 10 days could contribute to injury recovery by stimulating the translation of other neurexin isoforms.

The distruption of the neuropillin receptor system could upset the balance of semaphorin and VEGF signaling necessary to maintain the glomerular filtration barrier [40]. Plexin A1 is an important component of the neuropillin receptor complex and a known target of the three altered miRNAs studied in the present work (20b-5p, 347 and 466b). No clear pattern was observed in the expressions of these miRNAs. As for instance, miR-20b-5p was upregulated by DOR agonist in the 10-day group, while miRs-347 and 466b were suppressed under the same conditions. In contrast, DOR activation significantly upregulated miR-466b expression in the hypoxic kidney. Further investigation is required to determine a role for miRNA regulation of semaphoring signaling in hypoxic/ischemic kidney.

Hypoxic rats were unable to maintain the body and kidney weight gains as opposed to the control animals, concurrent to our previous observations [8], [41]–[43]. This reflects a mismatch between energy production and consumption under hypoxic conditions. Interestingly, the reductions in kidney/body weight ratio appeared earlier in the DOR-treated kidneys when compared to controls. Since opioid agents may affect feeding and appetite [44], [45], it is likely that UFP-512 influences the animal’s feeding through a DOR signaling pathway.

In summary, hypoxia comprehensively alters miRNA expression in the kidney with differential changes in various miRNAs depending on the duration of hypoxia. The altered miRNAs are known to target the antioxidant, apoptotic, survival and may other pathways. DOR activation has a significant effect on the miRNAome in the kidney under both normoxic and hypoxic conditions, leading to an improved condition for renal survival and functioning, though it does not improve the rate of body weight gain under hypoxia. MicroRNAs mediate mRNA suppression by recruiting miRNA-induced silencing complex (miRISC). The core of miRISC contains miRNA-loaded Argonature proteins (AGOs) and GW182. Since DOR activation greatly influences the miRNA profiling under hypoxic condition, the activity of AGOs and GW182 could be changed in response to hypoxia and DOR activation and needs to be further investigated. Moreover, it is important to explore if DOR inactivation with its antagonists influences the hypoxia and/or DOR activation induced changes in miRNAs. Further illumination of the targeted pathways associated with hypoxic kidney injury and the mechanism of DOR activation and the underlying mechanism may provide new insights into the potential therapeutics for hypoxic/ischemic injury of the kidney.

## Materials and Methods

### Animals

The experiments were approved by the Review Board and the Animal Care and Use Committee of Shanghai Research Center for Acupuncture and Meridians, and were performed in accordance with its guidelines. Male Sprague Dawley Rats were purchased from Shanghai Experimental Animal Center of Chinese Academy of Sciences. Twenty-one day old rats were randomly divided into 4 groups: (A) control, (B) DOR agonist (UFP-512), (C) chronic anoxia, and (D) chronic anoxia+DOR agonist (UFP-512). Groups A and B were raised in normal air and Groups C and D under hypoxic conditions.

### Induction of Chronic Hypoxia

Chronic hypoxia was induced as previously described [8], [41], [43], [46]. In brief, the hypoxic facility consisted of two parts: a plexiglass box (1.1 m×0.7 m×0.6 m) and an O_2_/CO_2_ analyzer to maintain an O_2_ level at 9.5%–10% in the box. The rats of Groups C and D were kept in the hypoxic box for 1, 5 or 10 days. The box was rapidly cleaned daily when animals were removed to record body weights.

### DOR Activation with UFP-512

The rats of Groups B and D were subjected to intraperitoneal injection of UFP-512 (H-Dmt-Tic-NH-CH[CH2-COOH]-Bid), a specific and potent DOR agonist synthesized by our team [13]. The injections (1 mg/kg in <1 ml) were performed on day 0 (immediately before the beginning of the hypoxia), day 4 and day 8. As a control, Groups A and C received the same amount of saline.

### Tissue Collection

After 1, 5 or 10 days of hypoxia, the rats were decapitated after deep anesthesia. Their kidneys were rapidly removed, weighed, frozen in liquid nitrogen and stored at −80°C until use.

### RNA Extraction and Microarray Experiments

The expression profile of kidney miRNAs after chronic hypoxia has not been previously described. Therefore, microarrays were used to examine the expression of miRNAs in the kidney after 10 days of hypoxia. Tissue samples were removed from liquid nitrogen and total RNA was extracted using Trizol Reagent (Invitrogen, Carlsbad, CA, USA). RNA labeling and hybridization on miRNA microarray chips were performed as previously described [47], [48]. In brief, 50 µg of total RNA were purified using the mirVANA miRNA isolation kit (Ambion, Carlsbad, CA, USA) to enrich for the small RNA fraction. The purified RNA was labeled with Cy3 and hybridization was carried out on a miRNA microarray chip (CapitalBio Corp., Beijing, China) containing 509 probes in triplicate, corresponding to 435 human miRNA genes. Hybridization signals were detected and the scanned images were used for quantification as described previously [47], [48]. Data analysis employed a two class-paired analysis within SAM, a statistical method that calculates a score for each gene and therefore identifies genes that are significantly associated with an outcome variable, such as hypoxia exposure. For this analysis, a false discovery rate of <5% was selected. MicroRNAs were considered significantly altered only when they fulfilled three criteria: (1) mean fold change >2 or <0.5; (2) *q*-value = 0; and (3) SAM score >2 or <−2 [49], [50]. After SAM analysis selection, the data of 22 miRNAs were assessed using unsupervised hierarchical clustering. Predicted targets for altered miRNAs were identified by cross-referencing the online data-bases: microCosm, TargetScan and Pictar.

### Quantitative RT-PCR

Total RNA isolated as above was treated with RNase-free DNase using a standardized protocol. Assays to quantify the mature miRNA were conducted as described previously [51], . Briefly, 1 µg of total RNA was reverse-transcribed to cDNA using AMV reverse transcriptase and looped antisense primers. The mixture was incubated at 16° for 15 min, 42° for 60 min and 85° for 5 min to generate a library of miRNA cDNAs. SYBR Green real-time PCR was then performed using a standardized protocol with a sequence detection system (Model 7500, Applied Biosystems, Foster City, CA, USA). The primers are listed in Table 1. In each assay, 1 µl of cDNA (1∶50 dilution) was used for amplification. The reactions were incubated in a 96-well optical plate at 95° for 5 min, followed by 40 cycles consisting of a 15 s interval at 95° and a 1-min interval at 60°. All reactions were performed in triplicate. After the experiments were completed, the threshold cycle (Ct) values were determined using the default threshold settings and for analyzing the RT-PCR data. The relative amount of each gene to internal control was calculated by using the equation 2^−ΔCt^, in which ΔCt = Ct _miRNA_−Ct _U6_. To exclude extreme outliers, miRNAs with expression lower than a threshold (Ct_miRNA_−Ct _U6_<15, mean fold change >2 or <0.5) were eliminated. The retained Ct data were normalized, mean-centered and log2-transformed.

**Table 1:** Primer sequences used for quantitative RT-PCR.

miRNA	Forward Primer	Reverse Primer
rno-let-7d	ACACTCCAGCTGGGAGAGGTAGTAGGTTGC	CTCAACTGGTGTCGTGGAGTCGGCAATTCAGTTGAGAACTATGC
rno-let-7f	ACACTCCAGCTGGGTGAGGTAGTAGATTGT	CTCAACTGGTGTCGTGGAGTCGGCAATTCAGTTGAGAACTATAC
rno-mir-101b	ACACTCCAGCTGGGTACAGTACTGTGATA	CTCAACTGGTGTCGTGGAGTCGGCAATTCAGTTGAGTTCAGCTA
rno-miR-135a	ACACTCCAGCTGGGTATGGCTTTTTATTCCT	CTCAACTGGTGTCGTGGAGTCGGCAATTCAGTTGAGTCACATAG
rno-mir-186	ACACTCCAGCTGGGCAAAGAATTCTCCTTT	CTCAACTGGTGTCGTGGAGTCGGCAATTCAGTTGAGAGCCCAAA
rno-miR-187	ACACTCCAGCTGGGTCGTGTCTTGTGTTGC	CTCAACTGGTGTCGTGGAGTCGGCAATTCAGTTGAGCCGGCTGC
rno-miR-199a-3p	ACACTCCAGCTGGGACAGTAGTCTGCACAT	CTCAACTGGTGTCGTGGAGTCGGCAATTCAGTTGAGTAACCAAT
rno-miR-199a-5p	ACACTCCAGCTGGGCCCAGTGTTCAGACTAC	CTCAACTGGTGTCGTGGAGTCGGCAATTCAGTTGAGGAACAGGT
rno-miR-20b-3p	ACACTCCAGCTGGGACTGCAGTGTGAGCAC	CTCAACTGGTGTCGTGGAGTCGGCAATTCAGTTGAGCCAGAAGT
rno-miR-20b-5p	ACACTCCAGCTGGGCAAAGTGCTCATAGT	CTCAACTGGTGTCGTGGAGTCGGCAATTCAGTTGAGACCTGCAC
rno-miR-21	ACACTCCAGCTGGGTAGCTTATCAGACTGA	CTCAACTGGTGTCGTGGAGTCGGCAATTCAGTTGAGTCAACATC
rno-mir-212	ACACTCCAGCTGGGTAACAGTCTCCAGTCA	CTCAACTGGTGTCGTGGAGTCGGCAATTCAGTTGAGTGGCCGTG
rno-miR-25*	ACACTCCAGCTGGGAGGCGGAGACACGGGC	CTCAACTGGTGTCGTGGAGTCGGCAATTCAGTTGAGGCAATTGC
rno-miR-291a-5p	ACACTCCAGCTGGGCATCAAAGTGGAGGCC	CTCAACTGGTGTCGTGGAGTCGGCAATTCAGTTGAGAGAGAGGG
rno-miR-292-5p	ACACTCCAGCTGGGACTCAAACTGGGGGCT	CTCAACTGGTGTCGTGGAGTCGGCAATTCAGTTGAGCAAAAGAG
rno-mir-298	ACACTCCAGCTGGGGGCAGAGGAGGGCTGTT	CTCAACTGGTGTCGTGGAGTCGGCAATTCAGTTGAGGGGAAGAA
rno-miR-29a	ACACTCCAGCTGGGTAGCACCATCTGAAAT	CTCAACTGGTGTCGTGGAGTCGGCAATTCAGTTGAGTAACCGAT
rno-miR-29b	ACACTCCAGCTGGGTAGCACCATTTGAAATC	CTCAACTGGTGTCGTGGAGTCGGCAATTCAGTTGAGAACACTGA
rno-miR-31	ACACTCCAGCTGGGAGGCAAGATGCTGGCA	CTCAACTGGTGTCGTGGAGTCGGCAATTCAGTTGAGCAGCTATG
rno-mir-324-3p	ACACTCCAGCTGGGCCACTGCCCCAGGTGC	CTCAACTGGTGTCGTGGAGTCGGCAATTCAGTTGAGCCAGCAGC
rno-miR-347	ACACTCCAGCTGGGTGTCCCTCTGGGT	CTCAACTGGTGTCGTGGAGTCGGCAATTCAGTTGAGTGGGCGAC
rno-mir-351	ACACTCCAGCTGGGTCCCTGAGGAGCCCTTTGA	CTCAACTGGTGTCGTGGAGTCGGCAATTCAGTTGAGTCAGGCTC
rno-miR-363*	ACACTCCAGCTGGGCGGGTGGATCACGATG	CTCAACTGGTGTCGTGGAGTCGGCAATTCAGTTGAGAAATTGCA
rno-mir-365	ACACTCCAGCTGGGTAATGCCCCTAAAAAT	CTCAACTGGTGTCGTGGAGTCGGCAATTCAGTTGAGATAAGGAT
rno-mir-370	ACACTCCAGCTGGGGCCTGCTGGGGTGGAAC	CTCAACTGGTGTCGTGGAGTCGGCAATTCAGTTGAGAACCAGGT
rno-miR-431	ACACTCCAGCTGGGTGTCTTGCAGGCCGT	CTCAACTGGTGTCGTGGAGTCGGCAATTCAGTTGAGTGCATGAC
rno-mir-466b	ACACTCCAGCTGGGTATGTGTGTGTGTATG	CTCAACTGGTGTCGTGGAGTCGGCAATTCAGTTGAGCATGGACA
rno-miR-487b	ACACTCCAGCTGGGAATCGTACAGGGTCA	CTCAACTGGTGTCGTGGAGTCGGCAATTCAGTTGAGAGTGGATG
rno-mir-511	ACACTCCAGCTGGGATGCCTTTTGCTCTG	CTCAACTGGTGTCGTGGAGTCGGCAATTCAGTTGAGTGAGTGCA
rno-mir-615-5p	ACACTCCAGCTGGGGGGGGTCCCCGGTGCT	CTCAACTGGTGTCGTGGAGTCGGCAATTCAGTTGAGGATCCGAG
rno-miR-743a	ACACTCCAGCTGGGGAAAGACGCCAAACTG	CTCAACTGGTGTCGTGGAGTCGGCAATTCAGTTGAGTCTACCCA
U6	CTCGCTTCGGCAGCACA	AACGCTTCACGAATTTGCGT

### Statistical Analysis

Data are presented as mean ± SE with a minimum n = 3 for each group. The mean value of Group A for each time point is set equal to one. Statistical significance was determined using either a student’s t-test or a one-way ANOVA followed by Tukey’s Multiple Comparison Test on paired columns as appropriate.
